# COVID-19-Associated Mucormycosis Mimicking Odontogenic Pain: A Report of Three Cases

**DOI:** 10.1155/crid/8017426

**Published:** 2025-10-17

**Authors:** Pegah Sarraf, Mahboube Hasheminasab, Mehrfam Khoshkhounejad, Seyed Ali Abaee, Pardis akbari, Mohammad Hossein Nekoofar, Paul M. H. Dummer

**Affiliations:** ^1^Department of Endodontics, School of Dentistry, Tehran University of Medical Sciences, Tehran, Iran; ^2^Department of Oral and Maxillofacial Surgery, School of Dentistry, Tehran University of Medical Sciences, Tehran, Iran; ^3^Department of Orthodontics, University of the Pacific Dugoni School of Dentistry, San Francisco, California, USA; ^4^Department of Dental Biomaterials, School of Dentistry, Tehran University of Medical Sciences, Tehran, Iran; ^5^Dental Sciences, University of Santiago de Compostela, La Coruña, Spain; ^6^Oral Medicine, Oral Surgery and Implantology, University of Santiago de Compostela, La Coruña, Spain; ^7^School of Advanced Technologies in Medicine, Tehran University of Medical Sciences, Tehran, Iran; ^8^Department of Endodontic, Bahçeşehir University School of Dentistry, İstanbul, Türkiye; ^9^School of Dentistry, College of Biomedical and Life Sciences, Cardiff University, Cardiff, UK

**Keywords:** COVID-19, mucormycosis, odontogenic pain

## Abstract

**Aim:**

The aim of this study was to present three cases of COVID-19-related mucormycosis mimicking endodontic pain and to discuss the relevant dental and medical literature for this potential life-threatening disease.

**Summary:**

Mucormycosis (previously called zygomycosis) is a rare but serious infection caused by a group of fungi called mucormycetes. Mucormycosis mainly affects people who are medically compromised and have systemic health conditions or take drugs that lower their immune response. There has been an increase in case reports/series of mucormycosis in individuals diagnosed with COVID-19. This is likely attributable to the high doses and prolonged use of corticosteroids in the treatment of hospitalized COVID-19 patients. This increase in the prevalence of the condition is of importance, primarily because the fatality rate of patients with mucormycosis is high. Moreover, rapid growth and dissemination into soft tissue and bone with angioinvasion of the fungus are common, and delays in diagnosis can be fatal. This report describes three cases of mucormycosis that were associated with COVID-19 and presented with symptoms that mimicked odontogenic pain.

**Summary**

• Dentists should be aware of the predisposing factors of mucormycosis.
• Oral manifestations and radiographic appearances of the disease must be understood by clinicians.
• Any discrepancy between the patient's symptoms and the clinical findings should be carefully evaluated in order to arrive at an accurate diagnosis.
• In most cases of mucormycosis, a multidisciplinary approach to developing an effective plan to manage the condition is necessary.

## 1. Introduction

In 2019, the global population was exposed to an outbreak of COVID-19. During the pandemic, a substantial proportion of infected individuals were hospitalized as they required corticosteroid [1] and antibiotic therapy [2], oxygen (O_2_) support, with many requiring intubation and mechanical ventilation. Many patients suffered a cytokine storm [3], hyperglycemia, metabolic acidosis, elevated blood iron concentrations, and impaired phagocytic activity that predisposed them to fungal infections caused by microorganisms such as mucormycetes [2].

The increasing use of immunosuppressants to control severe complications of COVID-19 in hospitalized patients resulted in a substantial rise in the prevalence and incidence of mucormycosis [4], a rare fungal infection with a rapid course and high mortality rate that results in severe complications and sequelae, especially in the face [4]. It is currently the third most common fungal infection after aspergillosis and candidiasis [5] and is more common in immunocompromised individuals [4].

The number of reported cases of mucormycosis is greater in the maxilla than in the mandible, which has been attributed to the more prolific blood flow and greater vascular supply associated with the upper jaw [6]. Due to the high prevalence of mucormycosis in the head, neck, and oral cavity, dentists may be the first healthcare providers to detect the condition, and therefore, they have a key role in its early diagnosis [4].

Mucormycosis is an opportunistic fungal infection that can present in acute and invasive forms. Factors such as uncontrolled diabetes and immunodeficiency increase the risk of infection [7]. Mucormycosis is caused by saprophytic fungi such as *Rhizopus*, *Mucor*, *Cunninghamella*, *Rhizomucor*, *Saksenaea*, *Apophysomyces*, or *Lichtheimia* [8]. The condition is categorized into six types based on the anatomical location of its occurrence and the associated clinical symptoms. The prevalence of mucormycosis, listed from the most common to the least common, is as follows:

1. Rhino-orbito-cerebral mucormycosis (ROCM)
2. Pulmonary mucormycosis
3. Cutaneous mucormycosis
4. Gastrointestinal mucormycosis
5. Disseminated mucormycosis
6. Miscellaneous mucormycosis

ROCM is frequently observed in diabetic patients, with or without ketoacidosis [9]. In patients with ketoacidosis, elevated levels of serum glucose and deteriorating metabolic state contribute to the growth and proliferation of the fungus. Such patients often present with a range of signs and symptoms, including periorbital facial pain, swelling of the eyelids, proptosis, bilateral maxillary sinusitis, headache, and maxillary toothache [10].

In immunocompromised patients, the hyphae of the microorganisms associated with ROCM grow within the nasal cavity, extend toward the paranasal sinuses, and orbit and attack the blood vessels (angioinvasion). The vascular supply and phagocytic dysfunction increase the level of hyphae in blood vessels; after which, the microorganisms attack the vessels and cause purulent arteritis and vascular thrombosis, resulting in tissue infarction and subsequent necrosis [8].

Additionally, local hypoxia and low pH, diabetes or hyperglycemia due to steroid use, reduction of chemotactic factors, and reduction of phagocytic activity will create a suitable environment for the growth of fungal hyphae [6].

In this report, three immunocompetent patients with a history of hospitalization due to COVID-19 and taking high-dose corticosteroids are described. All patients were seen by a dentist for their chief complaint of severe dental pain. However, due to a lack of improvement in symptoms, they were subsequently referred to the Endodontic Department at the Dental School of Tehran University of Medical Sciences for diagnosis and management.

**Figure 1:**
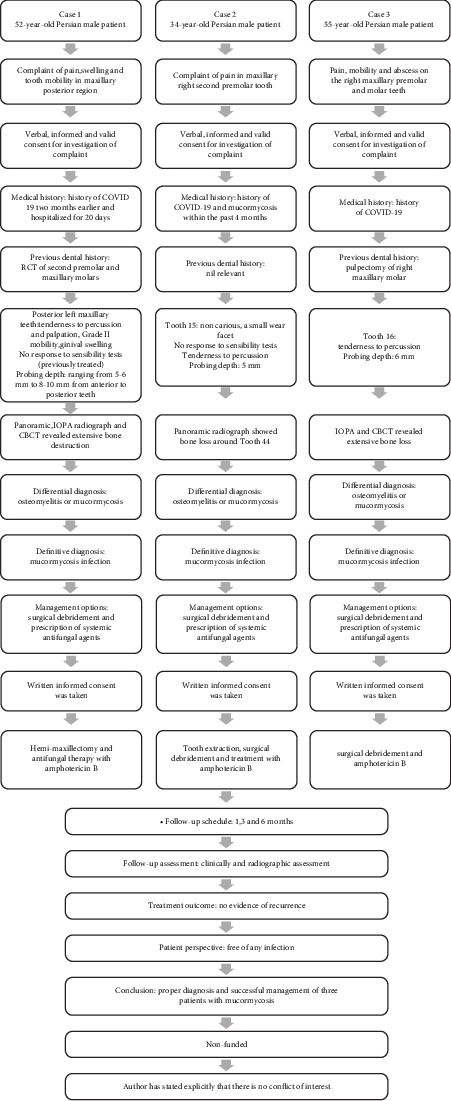
PRICE 2020 flowchart.

This case report was prepared according to the PRICE 2020 guidelines (Figure 1) [11].

## 2. Case 1

A 52-year-old Persian male was referred to the Department of Endodontics, Tehran University of Medical Sciences, complaining of loose posterior teeth on the left side of the maxilla and swollen gums in the same region. Upon preliminary examination and following the medical history, it was revealed that the patient had a history of COVID-19 2 months earlier. The patient had been hospitalized for 20 days after the definite diagnosis of COVID-19 and had 70% pulmonary involvement. He had been admitted to an intensive care unit for several days. The patient did not have any underlying systemic disease.

To address the symptoms of COVID-19, a range of broad-spectrum antibacterial and antiviral drugs, analgesics, and corticosteroids was prescribed to the patient during his 20-day hospitalization period (Table 1).

**Figure 2:**
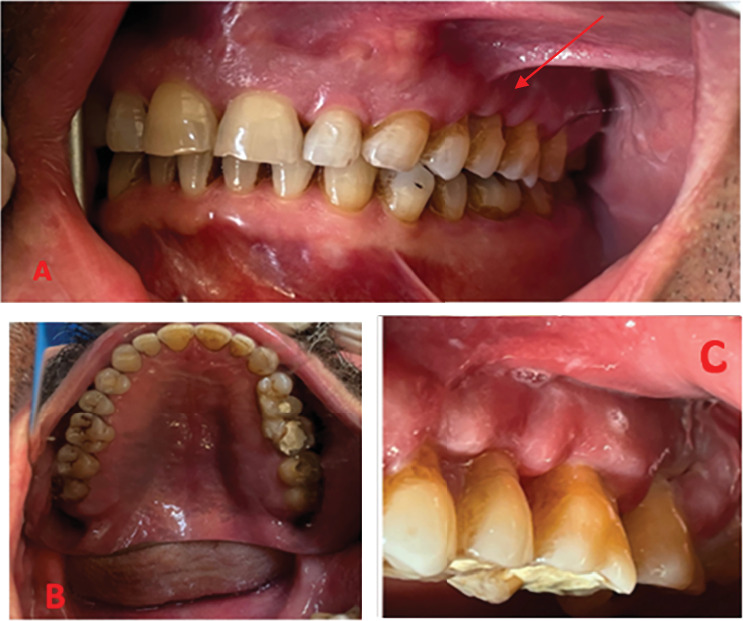
(A) Intraoral (frontal) clinical photograph of patient with gingival swelling, inflammation, and recession in the maxillary left quadrant. (B) Intraoral (palatal) clinical photograph of patient with left palatal swelling. Notice the teeth with temporary restorations following root canal treatment. (C) Close-up view of the abnormal soft tissue swelling in the maxillary left region.

Several days after being discharged from the hospital, the patient visited a dental clinic complaining of pain on the left side of his maxilla. Despite the teeth appearing intact on both radiographic and clinical examinations, the patient underwent root canal treatment of his second premolar and all three maxillary molars. Following root canal treatment, the patient experienced some relief from pain. However, approximately 20 days later, the patient noticed severe swelling and an abscess on the left side of his maxilla along with tooth mobility; he was referred to the department of endodontics to investigate the underlying cause of his signs and symptoms (Figure 2).

**Figure 3:**
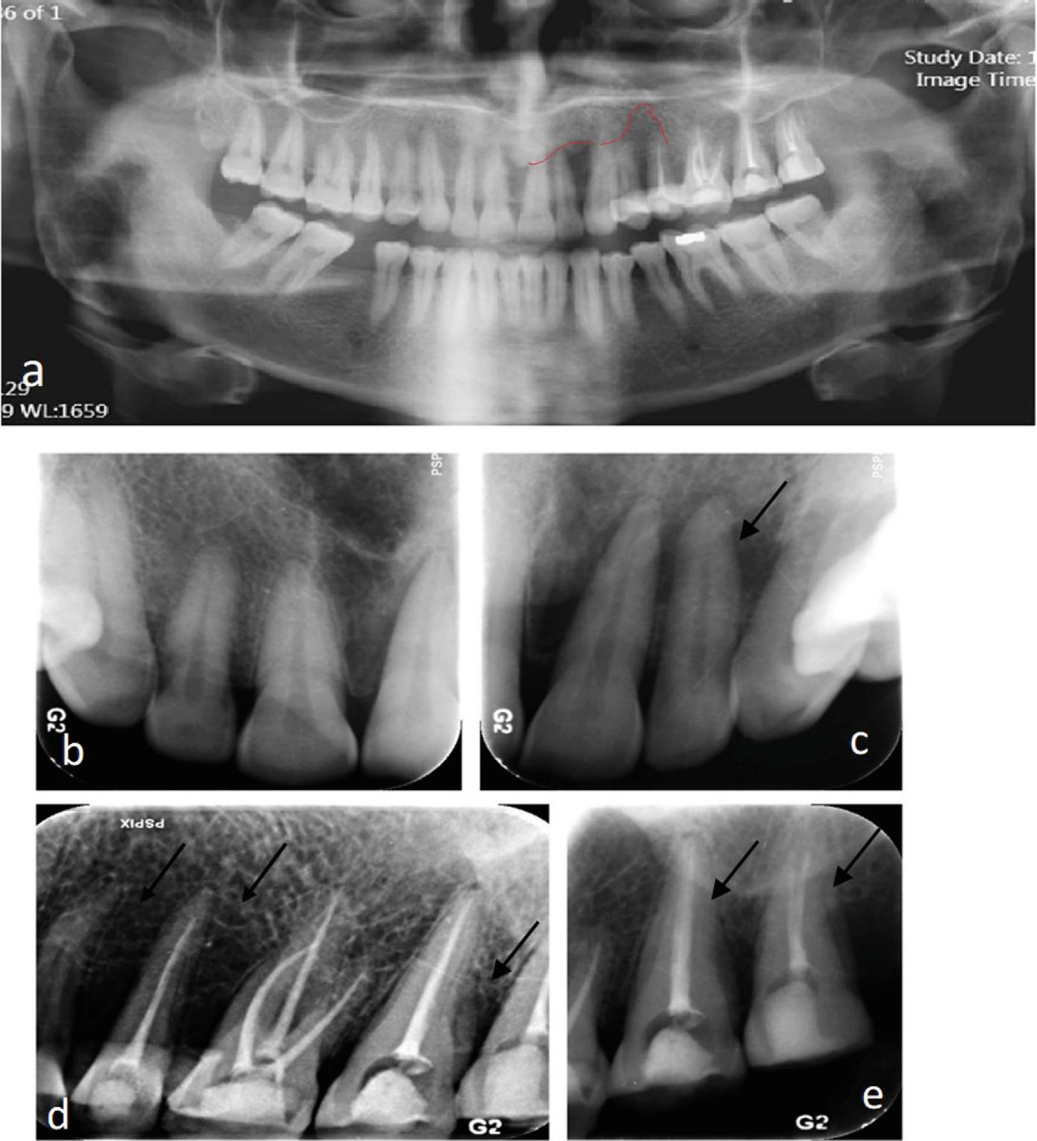
(a) Panoramic radiograph showing bone destruction in the right maxillary quadrant. (b) Periapical view of the right maxillary anterior teeth. (c) Periapical view of the left maxillary anterior teeth: the arrows show the bone resorption around Teeth 21 and 22. (d, e) Periapical radiographs of the left maxillary region: the arrows show the severe bone destruction around the root-filled maxillary premolars to the third molar.

**Figure 4:**
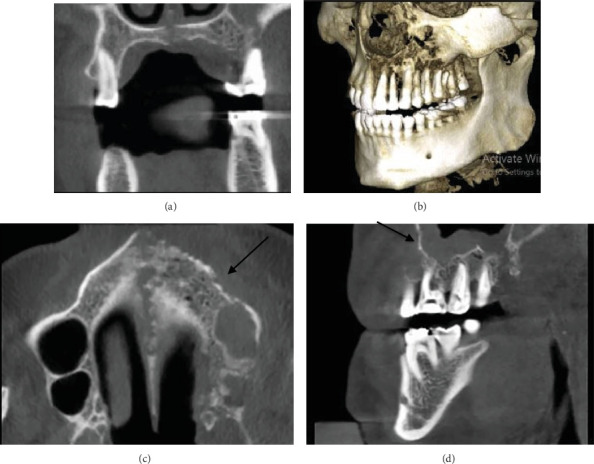
(a) Coronal CBCT view showing bone destruction, perforation of palatal bone, and obvious swelling of soft tissues in the left maxilla. (b) The reconstructed 3D image demonstrates the destruction of the bone and right maxillary sinus. (c) Axial CBCT view showing the involvement of the nasal bone and left sinus. The arrow shows the cortical bone destruction. (d) Sagittal CBCT view with the arrow showing the mucosal thickening of the sinus and bone destruction around the molar teeth.

Intraoral examinations revealed gingival swelling and Grade 2 mobility of all teeth in the left maxilla. The teeth were also sensitive to percussion and palpation. The teeth did not respond to sensitivity tests (second premolar to third molar teeth had been root filled previously). Probing depth measurements indicated a range of 5–6 mm in the area from the central incisor to the canine teeth and 8–10 mm in the area from the first premolar to third molar teeth. A panoramic radiograph revealed varying degrees of bone density, particularly in the anterior region, along with evidence of the destruction of the floor of the maxillary sinus. Periapical radiographs revealed apical bone loss around several teeth. The crowns of the teeth were intact apart from the access cavity preparations (Figure 3). A cone-beam computed tomography (CBCT) scan revealed severe bone destruction around the roots of the teeth in the left maxillary quadrant, presence of bone sequestrations, sphenoid bone involvement, sinus floor perforation, and loss of bone crest in the same quadrant. Furthermore, there was a noticeable contrast in the appearance of bone between the affected and unaffected sides, indicating a significant disparity in terms of the degree of bone destruction (Figure 4).

Differential diagnoses included osteomyelitis and mucormycosis. A maxillofacial surgeon was consulted, and the patient underwent an incisional biopsy from the maxillary sinus area, performed by an ENT specialist. The histopathological analysis confirmed the diagnosis of mucormycosis. Subsequently, the patient underwent hemimaxillectomy and received antifungal therapy with amphotericin B. Following a 1-month hospital stay, the patient was discharged and placed under regular follow-up care.

## 3. Case 2

**Figure 5:**
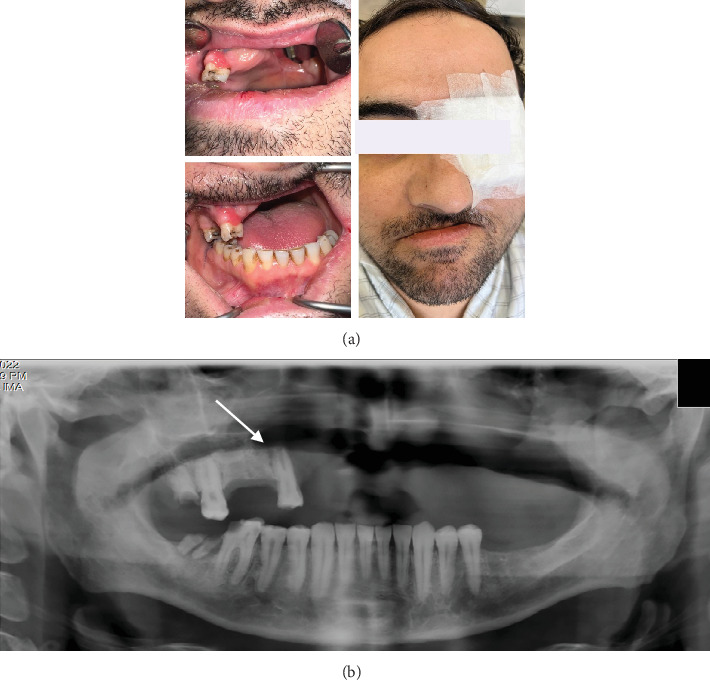
(a) Intraoral and extraoral photographs of the patient who had a history of hemimaxillectomy. The left orbit was exenterated due to the spread of the fungal infection. (b) Panoramic radiograph with the arrow pointing to bone destruction around the right maxillary premolar, suggesting recurrence of the disease.

A 34-year-old Persian male patient, who had contracted COVID-19 within the past 4 months and had undergone a hemimaxillectomy procedure along with the removal of part of his zygomatic bone and involvement of his right eye due to mucormycosis, was referred to the department of endodontics. The patient had no history of systemic diseases prior to COVID-19. The patient's chief complaint was pain in his maxillary right second premolar tooth. The tooth had a small wear facet and caries with no response to sensitivity tests (Figure 5); however, the tooth was tender to percussion. The periodontal probing depth was 5 mm in the mesial area at the incisional border of the previous surgical procedure. Severe bone loss was also observed in this region. Considering the history of mucormycosis and the chance of recurrence, the patient was referred to the department of oral and maxillofacial surgery. As part of the diagnostic process, an incisional biopsy sample was obtained. Histopathological examination revealed the recurrence of mucormycosis. Thus, the area was debrided, the tooth was extracted, and treatment with amphotericin B was initiated.

## 4. Case 3

**Figure 6:**
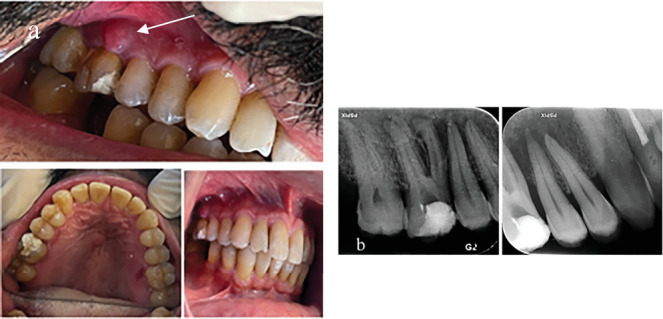
(a) Clinical images of the posterior maxillary quadrant; the arrow shows the swelling and fistula formation around the first molar. (b) Periapical radiographs showing bone destruction around the maxillary first molar and premolar teeth.

A 55-year-old Persian male patient, who had a confirmed history of COVID-19 approximately 3 months previously, was referred to the department of endodontics. The patient had no previous history of hospitalization except for COVID-19, during which he received a course of eight dexamethasone injections. The patient's chief complaints were pain, mobility, and abscess of the right maxillary molar and premolar teeth (Figure 6). The right maxillary molar had undergone an emergency pulpectomy despite having an intact crown, but 2 weeks later, the patient's symptoms had not resolved. The tooth was sensitive to percussion and had a periodontal probing depth of 6 mm. Radiographic examination revealed bone loss at the site of the right first molar and premolar teeth.

**Figure 7:**
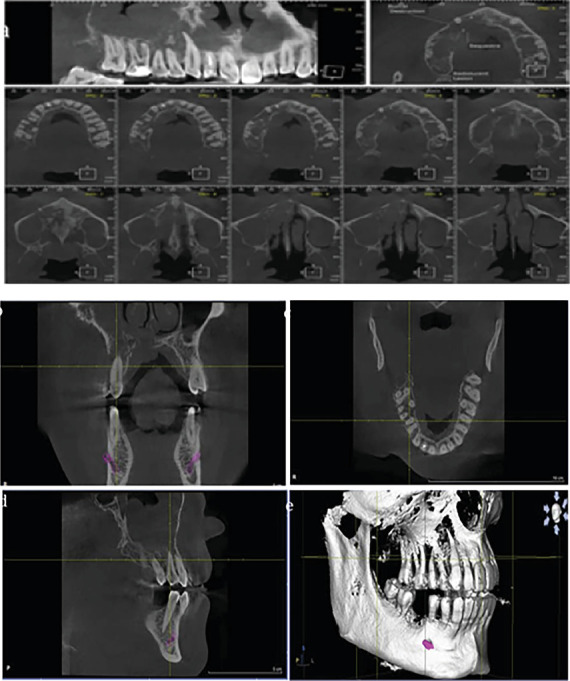
(a–e) Axial, sagittal, and coronal CBCT images and 3D reconstructed views showing severe bone destruction and sequestration.

A CBCT scan was requested which revealed extensive bone destruction in the right side of the maxilla and the sinus (Figure 7). Histopathological examination confirmed the diagnosis of mucormycosis. The patient underwent surgical debridement of the infected tissues, and amphotericin B was then prescribed. The patient was discharged from the hospital after 1 month and was placed under follow-up.

## 5. Discussion

Following the initial encounter and relying solely on subjective findings in these three cases, it seems plausible that the patients had lesions of endodontic origin. However, the unexplained diffuse pain and extensive bone destruction involving several intact teeth, even with a negative pulp sensibility test, are far more likely to be related to a nonodontogenic cause of the signs and symptoms. Cases 1 and 3 were misdiagnosed as endodontic problems, and unnecessary dental treatments were performed. As a consequence, there was an unnecessary delay in screening the patients for the true diagnosis, which may have had an impact on the radical nature of the treatment and the outcome.

There are several reports of dental pain as a consequence of mucormycosis [12, 13], mainly in patients with a history of diabetes or other systemic problems. The three patients in this report with symptoms mimicking odontogenic pain were referred to the endodontic department during the pandemic and did not have a history of systemic disease; the only common finding among them was a history of COVID-19 and respiratory failure, hospitalization, and extensive immunosuppressive therapy.

COVID-19 is a viral disease with multiorgan involvement that affects almost all vital systems of the human body. In patients admitted to hospital due to COVID-19, vascular thromboembolism occurs commonly due to immobility, dehydration, and cytokine storms. Management of nonambulatory patients with hormones and immunoglobulins can lead to an increase in vascular viscosity. Furthermore, vascular endothelial damage results in vascular destruction, eventually facilitating further spread of the disease and an increase in fungal growth [2, 14–17].

Prior to the onset of the COVID-19 pandemic, mucormycosis outbreaks primarily affected immunocompromised individuals, including those with diabetes mellitus, neutropenia, hematological malignancies, and other conditions that weaken the immune system [10].  Table 2 sets out the known predisposing factors for mucormycosis.

Geographical regions with hot and humid climates are an important risk factor for the spread of this fungal infection [4]. *Rhizopus arrhizus* is the causative agent of mucormycosis associated with COVID-19, which is also known as the black fungus, due to necrosis of the infected tissues [18]. In general, the mortality rate of mucormycosis infection in adults ranges from 20 to 100% depending on various factors such as the location and extent of the infection, treatability, presence of comorbidities, and the quality of healthcare available. The mortality rate of mucormycosis reported in children is reported to be approximately 33% [10]. Compromised immunity following corticosteroid therapy, long-term hospitalization, mechanical ventilation, lactic acidosis, increased ferritin, development of pseudodiabetic conditions due to increased expression of angiotensin-converting enzyme 2 receptor in pancreatic islets, increased insulin resistance due to cytokine storm, and vascular endothelial destruction due to COVID-19 all contribute to an increased mortality rate in patients with simultaneous mucormycosis and COVID-19 [17, 19].

Symptoms such as a toothache or localized intraoral swelling encourage patients to visit a dentist [4]. Gingival involvement accompanied by bone destruction and tooth mobility is among the common oral signs of mucormycosis as seen in the patients described in this report [4]. The most common sites of involvement in the oral cavity include the palate, gingiva, lips, and alveolar ridge. Mucormycosis can also spread through an oral mucosal tear or a wound after tooth extraction, as these sites are particularly susceptible to infection [19].

Early diagnosis, aggressive debridement of necrotic tissues, and prescription of appropriate systemic antifungal agents affect the survival rate [20]. Other adjunct treatments of mucormycosis include administration of pegfilgrastim or filgrastim colony–stimulating factors and recombinant cytokines, transfusion of granulocytes, and application of hyperbaric O_2_ (hyperbaric O_2_ therapy) [4]. Hyperbaric O_2_ therapy increases the level of O_2_ saturation, improves vascularity, induces the secretion of cytokines, and stimulates angiogenesis [7].

Mucormycosis is known to have a high recurrence rate, meaning that even after a successful initial treatment, there is a possibility of relapse, particularly during neutropenic periods or during antineoplastic chemotherapy [21, 22]. Therefore, regular follow-ups are equally as important as appropriate surgery in achieving treatment success. Long-term effects of the disease are also an important factor when providing comprehensive care to patients [4].

## 6. Conclusion

Although the COVID-19 pandemic appears to be under control, instances of acute cases are still encountered. Therefore, dentists should always take a precise history regarding recent COVID-19 infections, related hospitalization, and corticosteroid therapy when they encounter patients complaining of diffuse dental pain and unexpected tooth mobility in order to rule out mucormycosis and avoid unnecessary dental treatments. Patients who are suspected to have mucormycosis should be promptly referred for further examination and treatment to an appropriate medical specialist.

## Figures and Tables

**Table 1:** Medications prescribed during the hospitalization period for Case 1.

Azithromycin	500 mg cap.—Oral
Ceftriaxone	500 mg vial—IV
Cefepime	2 g vial—IV
Levofloxacin	Inj., 500 mg/20 mL, #2-IM
Remdesivir	Inj., 1 mg/5 mL, #7
Adalimumab (CinnoRA)	Inj., 0.8 mg/40 mL, #2
Tocilizumab (Actemra)	Vial, 0.9 mg/200 mL, #2
Dexamethasone	Amp., 2 mg/8 mL, #30
Aminophylline	Amp., 10 mg/250 mL, #2
Piroxicam	Amp., 1 mg/20 mL, #7

**Table 2:** Factors predisposing to mucormycosis [4].

**Systemic factors**	**Local factors**	**Others**
• Uncontrolled diabetes mellitus • Immunosuppression • Hematological malignancies • Bone marrow or organ transplantation • Chronic kidney disease (CKD) • COVID-19 • High serum iron concentrations • High serum ferritin and transferrin levels • Intravenous drug use • Malnutrition • Systemic steroid therapy • Antineoplastic chemotherapy • Deferoxamine use • Broad-spectrum antimicrobial therapy • Voriconazole use	• Chronic sinusitis • Burns • Tissue injuries including surgery	• Hot and humid climate • Use of nonhumidified oxygen • Improper sanitization of disposable humidifiers for oxygen administration • Reusing the same face mask (especially in humid environments)

## Data Availability

The data that support the findings of this study are available on request from the corresponding author. The data are not publicly available due to their containing information that could compromise the privacy of research participants.
